# Improving Cerebrovascular Function to Increase Neuronal Recovery in Neurodegeneration Associated to Cardiovascular Disease

**DOI:** 10.3389/fcell.2020.00053

**Published:** 2020-02-07

**Authors:** Lotte Vanherle, Hana Matuskova, Nicholas Don-Doncow, Franziska E. Uhl, Anja Meissner

**Affiliations:** ^1^Department of Experimental Medical Science, Lund University, Lund, Sweden; ^2^Wallenberg Centre for Molecular Medicine, Lund University, Lund, Sweden; ^3^Department of Neurology, University Hospital Bonn, Bonn, Germany; ^4^German Center for Neurodegenerative Diseases (DZNE), Bonn, Germany

**Keywords:** cerebral blood flow, cerebrovascular function, hypertension, heart failure, stroke

## Abstract

Mounting evidence indicates that the presence of cardiovascular disease (CVD) and risk factors elevates the incidence of cognitive impairment (CI) and dementia. CVD and associated decline in cardiovascular function can impair cerebral blood flow (CBF) regulation, leading to the disruption of oxygen and nutrient supply in the brain where limited intracellular energy storage capacity critically depends on CBF to sustain proper neuronal functioning. During hypertension and acute as well as chronic CVD, cerebral hypoperfusion and impaired cerebrovascular function are often associated with neurodegeneration and can lead to CI and dementia. Currently, all forms of neurodegeneration associated to CVD lack effective treatments, which highlights the need to better understand specific mechanisms linking cerebrovascular dysfunction and CBF deficits to neurodegeneration. In this review, we discuss vascular targets that have already shown attenuation of neurodegeneration or CI associated to hypertension, heart failure (HF) and stroke by improving cerebrovascular function or CBF deficits.

## Introduction

The cerebral circulation plays a critical role in matching nutrient and oxygen supply to neuronal activity and thus, is intimately linked to proper brain function (Iadecola, 2013, 2017; Wolters et al., 2017; Leeuwis et al., 2018). However, it appears to have been long under-recognized in the field of neurodegenerative research despite mounting evidence that associates also classical neurodegenerative diseases, such as Alzheimer’s disease and Parkinson’s disease, to impairments in cerebrovascular structure and function (Freitag et al., 2006; Kovacs et al., 2014; Bos et al., 2017). We have come a long way, and focus has shifted to include the vascular origins of neurodegeneration (Iadecola, 2013; Castillo et al., 2019).

In recent years, it came apparent that during cardiovascular disease (CVD), the most prevalent disease burden worldwide (GBD 2017 Causes of Death Collaborators, 2018), structural and functional impairments of the cerebral circulation majorly contribute to the development of neurodegeneration and cognitive impairment (CI) (Kuller et al., 2005; Jefferson et al., 2010; Gorelick et al., 2011; Haring et al., 2013; Takeda et al., 2019). Cerebrovascular alterations, ranging from endothelial dysfunction, vascular remodeling and inflammation to capillary rarefaction, blood-brain-barrier (BBB) damage and neurovascular uncoupling promote neurodegeneration in CVD (Iadecola, 2013, 2017) and have also been associated to the pathogenesis of Alzheimer’s disease (Carnevale et al., 2016). Precise mechanisms underlying neurodegenerative processes and CI in CVD are reviewed in detail elsewhere (Iadecola, 2013; O’Brien and Thomas, 2015; Castillo et al., 2019), but it is generally considered that such cerebromicrovascular alterations contribute to a decline in cerebral blood flow (CBF) that reduces metabolic support for neural signaling, thereby exacerbating neuronal dysfunction (Iadecola, 2013). Strong epidemiological and experimental evidence suggest an exacerbation of cognitive dysfunction during *hypertension* and *heart failure* (HF) (Jefferson et al., 2010; Gorelick et al., 2011). Thus, controlling cardiovascular risk factors has become increasingly important not only in the prevention of deleterious acute consequences (i.e., *stroke*), but also for reducing the risk of developing CVD-associated neurodegeneration that may lead to the development of CI and dementia (Sprint Mind Investigators for the Sprint Research Group, Williamson et al., 2019). Although the association between CVD and increased dementia risk is well established (Kuller et al., 2005; Jefferson et al., 2010; Gorelick et al., 2011; Haring et al., 2013), it is still unclear if treatments would reverse already established CI. Because of its intimate link to neuronal function, targeting the cerebral vasculature to improve CBF has yielded some promising results in respect to neuro-regenerative processes (Meissner et al., 2012; Lidington et al., 2019). This article summarizes vascular targets that have shown to attenuate neuronal degeneration or cognitive function associated to hypertension, HF and stroke by improving cerebrovascular function or CBF deficits. For this purpose, we mainly included studies in this review that discussed mechanisms related to cerebrovascular function or CBF regulation, which showed improvements of vascular function and/or a mitigation of neurodegeneration (e.g., impairment of neuronal structure, memory function, neurological function) after targeting these mechanisms. We apologize to all researchers whose work is only indirectly mentioned through review article citations.

## Targeting Cerebrovascular Mechanisms to Improve Cbf and Attenuate Neurodegeneration During Hypertension and Cardiovascular Disease

### Hypertension

Chronic hypertension is the most prevalent cardiovascular disorder and the leading cause of cardiovascular and cerebrovascular morbidity and mortality worldwide (Drozdz and Kawecka-Jaszcz, 2014; Ibekwe, 2015). It is one of the most important modifiable risk factors for stroke and HF (Ibekwe, 2015), and has also been associated to the pathogenesis of Alzheimer’s disease (Carnevale et al., 2016). Hypertension alters the morphology as well as the function of cerebral vessels (Joutel et al., 2010; Meissner, 2016; Meissner et al., 2017), however, our knowledge about its effects on neurovascular coupling and hypertension-induced molecular changes in the different cell types of the neurovascular unit is still very fragmented.

Angiotensin II (AngII), the primary effector hormone of the renin angiotensin system (RAS), is considered the main contributor to impairments of neurovascular coupling independent of blood pressure (Kazama et al., 2004). It is suggested that vascular and glial cells, but not neurons, drive the changes in neurovascular coupling in response to AngII (Kazama et al., 2004; Capone et al., 2011). In the group of hypertensive drugs, angiotensin II receptor type 1 (AT1R) antagonists have been shown to prevent a decline in CBF in elderly hypertensive patients (Bloch et al., 2015) by potentially counteracting cerebrovascular dysfunction and local reactive oxygen species (ROS) production. Interestingly, treatment with Ang-(1-7), which are opposing many AngII effects on AT1R, lowered AngII levels in spontaneously hypertensive rats (SHR) and most interestingly, associated to decreased neuronal apoptosis. Favorable Ang-(1-7) effects on CBF are thought to result from effects on bradykinin levels (Lu et al., 2008), nitric oxide (NO) release, and endothelial NO synthase (NOS) expression (Zhang et al., 2008), and through Mas receptors (Jiang et al., 2013) that are thought to be involved in astrocyte-mediated calcium signaling during hypertension (Guo et al., 2010).

Recent evidence has emerged that statins, cholesterol-lowering drugs, may be beneficial in hypertension, specifically for the reversal of cognitive decline (Don-Doncow et al., 2018). Hypertension-induced BBB impairment was reversed after atorvastatin treatment, suggesting a novel role for statins in hypertension-associated brain dysfunction (Kalayci et al., 2005). Moreover, simvastatin normalized cerebromicrovascular perfusion and increased cerebral capillary density in SHR (Freitas et al., 2017). Precise mechanisms underlying these favorable effects on the cerebral microvasculature are yet to be determined. Besides affecting cerebromicrovascular perfusion and BBB stability, statin-mediated anti-inflammatory effects decreased in both rolling leukocyte presence and leukocyte adhesion to cerebral endothelial cells (Freitas et al., 2017), which may hinder leukocyte infiltration into the brain and thus, reduce neuroinflammation (Don-Doncow et al., 2018). Adverse immune activation and infiltration is considered a key mechanism in neurodegeneration associated to hypertension or hypertensive stimuli (Faraco et al., 2016, 2018; Don-Doncow et al., 2019). High dietary salt concentrations have been shown to suppress endothelial function and CBF, resulting in CI by mechanisms involving an expansion of the Th17 cells in the small intestine that leads to a systemic increase in IL-17 (Faraco et al., 2018). By inhibiting the phosphorylation of endothelial NOS and thus, NO production in cerebral endothelial cells, IL-17 is thought to contribute to the neurovascular dysfunction and thus, the development of CI (Faraco et al., 2018). Neuroinflammation is generally accepted as a key player in the pathophysiology of hypertension, involving higher oxidative stress levels, immune activation and recruitment, and BBB impairment (Rodriguez-Iturbe et al., 2017). To date, only little is known about the precise mechanisms linking hypertension-associated inflammation to cerebrovascular dysfunction or CBF and hence, its link to neurodegeneration and CI is mostly elusive. A recent study showed the role of perivascular macrophages (PVMs) as mediators of hypertension-associated neuronal dysfunction and memory impairment in response to AngII (Faraco et al., 2016). The activation of the AT1R on PVMs, which was shown to induce NADPH oxidase 2 (NOX2)-mediated ROS production, is not only thought to trigger BBB impairment but also CBF reduction. Depletion of PVMs as well as PVM-specific AT1R deletion protected against the development of neurovascular uncoupling, CBF deficits and memory impairment induced by hypertension (Faraco et al., 2016). Overexpression of CuZn-superoxide dismutase in the subfornical organ prevented hypertension-induced alteration in neurovascular coupling and endothelium-dependent responses in somatosensory cortex, confirming an AngII-mediated neurovascular unit dysfunction during hypertension that involves ROS (Capone et al., 2012). Despite proven mechanistic involvement, ROS inhibition or immunomodulation have not yet been evaluated therapeutically in hypertension-associated neurodegeneration. Like ROS, many other targets have been identified as mechanisms linking neurodegeneration and hypertension, but their effective therapeutic potential to promote neuro-regeneration in the hypertensive brain is yet to be tested.

### Heart Failure

Heart failure-associated morbidity has grown as the frequently accompanying cognitive decline majorly affects disease outcome, accelerating disease progression by reducing the ability to execute self-care activities and treatment compliance (Hajduk et al., 2013). Epidemiological studies not only showed that the majority of HF patients develop some form of cognitive decline or memory loss, but that they develop them earlier in life compared to the healthy population (Cacciatore et al., 1998; Pressler, 2008; Pressler et al., 2010; Hajduk et al., 2013; Leto and Feola, 2014; Meissner et al., 2015). To date, no therapeutic options exist. Restoring hemodynamic properties and correcting vascular risk factors seem to be the primary approach in the management of CI in HF patients. Experimental mouse models emulate several key features of HF-associated brain complications, including reduced CBF and compromised neurological function (Meissner et al., 2015), and are therefore valuable model systems to study molecular mechanisms underlying neurodegeneration and CI associated to HF.

**FIGURE 1:**
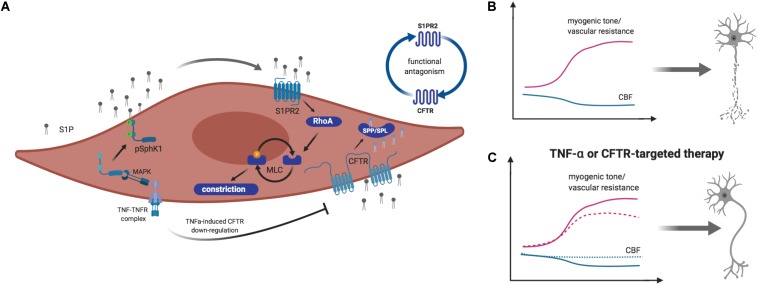
Schematic illustration of sphingosine-1-phosphate (S1P) signaling-mediated myogenic tone regulation in vascular smooth muscle cells. **(A)** Tumor necrosis factor alpha (TNF-α) stimulates sphingosine kinase 1 (Sphk1)-mediated S1P production in a mitogen activated protein kinase (MAPK)-dependent manner. S1P is released to the extracellular space, where it activates S1P receptor (S1PR) 2–dependent signaling pathways, leading to vasoconstriction. TNF-α stimulates the downregulation of the cystic fibrosis transmembrane conductance regulator (CFTR), which transports S1P across the plasma membrane for degradation by S1P phosphohydrolases (SPP) or S1P lyases (SPL). **(B)** During heart failure (HF) and hemorrhagic stroke, augmented S1P production by SphK1 and limited S1P degradation enhance S1PR2-mediated vasoconstriction that leads to reduced cerebral blood flow (CBF) and neurodegeneration. **(C)** Targeting S1PR2 signaling by inhibiting S1PR2 or improving S1P degradation by enhancing CFTR expression and activity normalizes myogenic tone and cerebral perfusion. This normalization associates to reduced neuronal injury. Image created by BioRender.

In healthy patients, cerebral auto-regulatory mechanisms can compensate for fluctuations in cardiac output and blood pressure by lowering cerebrovascular resistance (Paulson et al., 1990; Willie et al., 2014). During HF, however, increased vascular tone successes vasodilation that normally counterbalances increased vasoconstriction (Gruhn et al., 2001; Choi et al., 2006; Yang et al., 2012). Hence, cerebral autoregulation might not be able to fully compensate hemodynamic changes, leading to decreased CBF that may directly translate to structural and functional alterations in the brain (Yang et al., 2012). Previous work identified a novel microvascular mechanism by which HF critically reduces CBF and thus, impairs memory function (i.e., tested in a novel object recognition task) in a mouse model of congestive HF. Here, augmented sphingosine-1-phosphate (S1P) signaling associates to enhanced myogenic tone that translates into compromised autoregulation and restricted CBF (Meissner et al., 2012; Yang et al., 2012). Pharmacological inhibition as well as genetic deletion of S1P receptor 2 (S1PR2) abolished the HF-induced augmentation of myogenic tone in isolated posterior cerebral arteries (Yang et al., 2012). Such augmented cerebrovascular S1PR2 signaling results from disturbances in S1P homeostasis during HF where an acquired cystic fibrosis transmembrane regulator (CFTR) dysfunction critically impairs the cerebrovascular S1P degradation, and hence increases S1P bioavailability for S1PR2 signaling on vascular smooth muscle cells (Meissner et al., 2012). Augmented tumor necrosis factor alpha (TNF-α) signaling in cerebral arteries was identified as molecular link that not only stimulates S1P production but also limits S1P degradation by down-regulating CFTR (Meissner et al., 2012; Yang et al., 2012; Figure 1). Scavenging TNF-α with Etanercept successfully abolished the pathological augmentation of cerebrovascular vasoconstriction in HF and thus, improved cerebral perfusion (Meissner et al., 2015). As a clinical intervention, however, Etanercept carries significant unwanted risks associated to its importance in neuronal function and blood pressure control (Baune et al., 2008; Kroetsch et al., 2017). Similarly, directly targeting S1P and its receptors bears considerable disadvantages due to its pleiotropy and cell-type specific functionality. Thus, correcting S1P homeostasis by normalizing S1P homeostasis (i.e., through stabilizing CFTR function) yielded beneficial effects (Lidington et al., 2019). Pharmacological treatment of HF mice improved neuronal integrity (i.e., dendritic lengths and spine density) and memory function through normalizing pathological alterations in cerebral artery CFTR expression, vascular reactivity, and CBF (Lidington et al., 2019). Targeting S1P-CFTR signaling, therefore, may emerge as valuable tool to manage cerebrovascular dysfunction, impaired cerebral perfusion, and neuronal injury contributing to HF-associated neurodegeneration and memory deficits. CFTR therapeutics could have unanticipated, non-beneficial effects in other tissues by for instance, increasing CFTR expression above normal levels. Thus, repurposing of clinically approved CFTR correctors necessitates more rigorous pre-clinical testing.

Another target of pre-clinical investigation in attempt to link HF and neurodegeneration is inflammation associated to CBF deficits. In a mouse model of slowly developing HF, vascular CI was observed during the early stages of HF and interestingly, preceded the development of left ventricular dysfunction (Adamski et al., 2018). Precise non-cardiac-dependent contributors to such early onset vascular CI still need to be confirmed. However, platelet hyperactivity, contributing to BBB impairment and in turn, cerebral endothelial inflammation and impairment of NO−dependent vasoregulation was put forward to promote the development of CI and lead to a chronic manifestation of CBF deficits during advanced HF (Adamski et al., 2018). Key players involved in this signaling cascade remain unknown and hence, platelet-targeting therapy as strategy to reverse neurodegeneration and CI associated to early HF is yet to be validated. To date, only few studies have investigated molecular targets to re-establish normal CBF during HF, which highlights the need for more pre-clinical research efforts to identify targetable mechanisms with capacity to improve brain function during HF.

### Stroke

Stroke is associated with the highest incidence of severe disability, of which neurological deficits are reported in 50% of all patients 6 months after a stroke event (Bordet et al., 2017; Merriman et al., 2018). Ischemic stroke leads to complete blood flow disruption, resulting in irreversible changes and cell death in the ischemic core and in parts of the surrounding area, the so-called penumbra. Reperfusion in the penumbra in the acute phase after ischemia onset limits the extent of tissue damage and helps improving functional outcome. It is, therefore, the main target of acute neuroprotective treatments (Lin et al., 2013), of which tissue plasminogen activator (tPA) is currently the only approved medication. The therapeutic window of tPA, however, is limited to 3–4.5 h post-stroke onset as tPA administration beyond 4.5 h increases the risk of developing edema and hemorrhagic transformation that in turn, is associated with delayed ischemia (Pena et al., 2017). Targeting vascular function and re-establishing proper CBF has been focus of many preclinical research efforts and yielded some promising results that warrant testing in humans (Iwasawa et al., 2018; Lidington et al., 2019).

The stimulation of angiogenesis via the vascular endothelial growth factor (VEGF) represents one of the most researched targets to improve CBF after stroke despite the controversy regarding its effects. Studies using VEGF treatment post-stroke revealed a clear importance of administration route and timing. VEGF injected systematically in the acute post-stroke phase induces BBB leakage and hemorrhagic transformation (Abumiya et al., 2005) accompanied by augmented ischemic lesions. Intravenous injection of human recombinant VEGF 48 h post-stroke on the other hand promoted angiogenesis and reduced neurological deficit in rats (Zhang et al., 2000). Mice overexpressing VEGF post-stroke had smaller ischemic volume and more pronounced new vessel formation, interestingly, without CBF improvements in the ischemic area or aggravating effects on the BBB (Wang et al., 2005). In addition to angiogenesis, the beneficial effect of exogenous VEGF may also be mediated by its anti-apoptotic and pro-survival effects in neurons (Sanchez et al., 2010). Accordingly, brain derived neurotrophic factor (BDNF) was identified as major regulator of angiogenic processes (Usui et al., 2014) that mediates its angiogenic effects via a crosstalk with VEGF (Li et al., 2006). In stroke patients significant lower BDNF levels were detected, which were positively correlated with poor functional outcome (Schabitz et al., 2007). Similar to VEGF, exogenous BDNF administration post-stroke resulted in smaller ischemic size and significantly improved functional outcome in rat models by inducing hippocampal neurogenesis and reducing neuroinflammation in the acute phase of stroke (Schabitz et al., 2007; Ravina et al., 2018). Its effects on CBF related to improved stroke outcome, however, are still elusive. Nerve growth factor (NGF) seems to be similarly linked to VEGF-mediated pro-angiogenic effects in the ischemic brain. In rodent models, intranasal administration of NGF increased VEGF serum levels, increased microvessel density in the penumbra region, improved neurological outcome, and reduced ischemic injury 7 days post-stroke (Li et al., 2018). The beneficial effects of intranasal application of NGF are currently tested in a clinical trial in patients after acute ischemic stroke (ClinicalTrials.gov Identifier: NCT03686163). Although effects on CBF are warranted for most of these studies, angiogenesis-boosting therapy to re-establish proper perfusion evolved as promising therapeutic target in acute stroke therapy. Besides testing the classical growth factor-mediated therapy approaches in rodent models, AT1R blockers, which are widely used anti-hypertensive medications, were re-discovered to show pro-angiogenic effects after stroke and were reported to increase CBF in the ipsilateral hemisphere (Ito et al., 2002; Engelhorn et al., 2004; Alhusban et al., 2013). Similarly, AngII type 2 receptor (AT2R) agonists were shown to result in enhanced relaxation of basilar arteries and improved CBF after permanent middle cerebral artery occlusion (Faure et al., 2008). Nonetheless, the effectiveness of Angiotensin receptor (ATR) treatment is under debate as LIFE, ACCESS and MOSES trials demonstrated a decrease in the frequency of stroke after AT1R blockade (Dahlof et al., 2002; Schrader et al., 2003, 2005), while such treatment failed to show beneficial effects in other trials (Sandset et al., 2011).

Similar to cerebrovascular ATR effects, modulation of S1P receptors has shown beneficial effects on cerebrovascular function and CBF in experimental stroke. The selective S1PR1 agonist SEW2871 revealed positive effects on CBF, potentially resulting from increased diameter of leptomeningeal collateral vessels and enhanced vasodilatation in leptomeningeal anastomoses associated with increased phosphorylation of endothelial NOS in the ipsilateral hemisphere during chronic cerebral hypoperfusion (Iwasawa et al., 2018). Besides CBF improvements, researchers also reported an attenuation of infarct size and an improvement of neurological function assessed by neuroscoring.

In a model of subarachnoid hemorrhage (SAH), a form of stroke that originates from a hemorrhage but often exacerbates through secondary ischemia (van Gijn et al., 2007), increased cerebrovascular myogenic tone and hence, increased cerebrovascular resistance and reduced CBF was associated to augmented S1PR2 signaling in vascular smooth muscle cells (Yagi et al., 2015). Therapeutic administration of S1PR2 antagonist JTE013 improved cerebrovascular function, reduced neuronal injury and significantly enhanced neurological function (i.e., neuroscore reduction). TNF-α inhibition resulted in a similarly improved functional outcome (Yagi et al., 2015). TNF-α-induced impairment of cerebrovascular function that associates to CBF deficits and secondary ischemia during SAH (Vecchione et al., 2009; Yagi et al., 2015) are thought to stem from altered S1P signaling as TNF-α alters S1P degradation, which leaves more S1P available for pro-constrictive S1PR2 signaling in mural cells (Figure 1). Improving S1P degradation normalized the perfusion deficits, reduced neuronal injury, and improved neurological function in this SAH model (Lidington et al., 2019).

Overall, targeting the cerebral vasculature and improving vascular function and thus, normalizing CBF post-stroke evolved as promising path to improve post-stroke recovery. More studies are needed that evaluate CBF effects of different treatment strategies in addition to the commonly used assessment criteria for stroke outcome, such as infarct volume and neuroscore.

## Outlook

We have come a long way to appreciate the association between CVD and cognitive function. Hence, efforts to preserve and restore cerebrovascular function and integrity drastically increased. The identification of important drivers of vasoregulation and thus, CBF is more and more valued to possess the capacity as therapeutic intervention early in disease processes to either prevent neurodegeneration or boost neuro-regeneration not only in CVD but also in classical neurodegenerative diseases. However, our mechanistic knowledge relating CVD-associated cerebrovascular dysfunction to neurodegeneration and potentially CI is still very fragmented, with only few targets with larger applicability emerging thus far. Particularly, the modulation of AT1R or S1P signaling has shown promising effects on cerebrovascular function and CBF and in turn, neuronal function in different pre-clinical disease models (Meissner et al., 2012; Yang et al., 2012; Yagi et al., 2015). Other emerging targets with a wider applicability still warrant a validation in CVD models. Transcranial infrared brain stimulation (TIBS) is suggested to stabilize patients with CI and memory deficits through stimulation of mitochondrial ATP production in the brain (de la Torre et al., 2019). This approach may not only counteract reduced energy availability but also increase CBF through augmenting endothelial NO production and thereby, limit neuronal degeneration (Uozumi et al., 2010). Further research is needed to identify unifying mechanisms that are applicable to several diseases. In light of that, engagement of different pre-clinical and clinical communities in concerted efforts is required to successfully answering still outstanding question.

## Author Contributions

LV, HM, and AM wrote the first draft of the manuscript. ND-D and FU wrote sections of the manuscript. All authors contributed to the manuscript revision, read, and approved the submitted version.

## Conflict of Interest

The authors declare that the research was conducted in the absence of any commercial or financial relationships that could be construed as a potential conflict of interest.
